# Two‐Photon Polymerization of Nanocomposites for the Fabrication of Transparent Fused Silica Glass Microstructures

**DOI:** 10.1002/adma.202006341

**Published:** 2021-01-14

**Authors:** Frederik Kotz, Alexander S. Quick, Patrick Risch, Tanja Martin, Tobias Hoose, Michael Thiel, Dorothea Helmer, Bastian E. Rapp

**Affiliations:** ^1^ Glassomer GmbH Georges‐Köhler‐Allee 103 79110 Freiburg Germany; ^2^ Laboratory of Process Technology NeptunLab Department of Microsystems Engineering (IMTEK) University of Freiburg 79110 Freiburg Germany; ^3^ Freiburg Materials Research Center (FMF) University of Freiburg 79104 Freiburg Germany; ^4^ Nanoscribe GmbH Hermann‐von‐Helmholtz‐Platz 6 76344 Eggenstein‐Leopoldshafen Germany; ^5^ FIT Freiburg Centre of Interactive Materials and Bioinspired Technologies University of Freiburg 79110 Freiburg Germany

**Keywords:** 3D printing, direct laser writing, fused silica glass, nanocomposites, two‐photon polymerization

## Abstract

Fused silica glass is the material of choice for many high‐performance components in optics due to its high optical transparency combined with its high thermal, chemical, and mechanical stability. Especially, the generation of fused silica microstructures is of high interest for microoptical and biomedical applications. Direct laser writing (DLW) is a suitable technique for generating such devices, as it enables nearly arbitrary structuring down to the sub‐micrometer level. In this work, true 3D structuring of transparent fused silica glass using DLW with tens of micrometer resolution and a surface roughness of *R*_a_ ≈ 6 nm is demonstrated. The process uses a two‐photon curable silica nanocomposite resin that can be structured by DLW, with the printout being convertible to transparent fused silica glass via thermal debinding and sintering. This technology will enable a plethora of applications from next‐generation optics and photonics to microfluidic and biomedical applications with resolutions on the scale of tens of micrometers.

## Graphical abstract

High‐resolution 3D printing of photocurable silica nanocomposites using two‐photon direct laser writing is presented. The nanocomposites are consecutively debound and sintered to high‐quality fused silica microstructures with optical surface roughness.

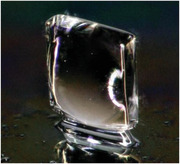

Transparent silicate glasses are one of the most important materials in advanced engineering applications including microsystems technology (MEMS). High‐optical transparency combined with a high thermal and chemical resistance are the key properties that make fused silica glass first choice for optics, photonics, microfluidics, and chemistry applications.^[^ ^1^, ^2^, ^3^ ^]^ While traditional techniques like grinding and polishing allow manufacturing of high‐precision macroscopic objects, they only allow simple geometries with micrometer resolution, e.g., V‐grooves.^[^ ^4^ ^]^ Another microstructuring method for fused silica glass is wet chemical etching, which is restricted mainly due to the isotropic nature.^[^ ^5^ ^]^ Laser‐assisted etching overcomes the restrictions of chemical etching.^[^ ^6^, ^7^ ^]^ However, the process results in rough surfaces, usually in the range of 40–200 nm which are noncompatible with optical applications and need substantial postprocessing.^[^ ^3^, ^8^ ^]^ As an alternative, 3D printing has recently emerged as a suitable technology for the fabrication of glass components. Direct printing, using fused deposition modeling approaches,^[^ ^9^, ^10^ ^]^ results in macroscopic glass components which are not capable of producing high‐resolution glass components. Indirect 3D printing technologies use glass precursors like nanocomposites^[^ ^11^, ^12^, ^13^, ^14^ ^]^ or sol‐gel mixtures, which can be printed using, e.g., stereolithography (SL) printing or direct ink writing.^[^ ^11^, ^15^, ^16^, ^17^ ^]^ The printed precursors are subsequently converted to glass via a heat treatment. Due to the layer‐based nature, these techniques fabricate parts with significant staircase defects showing the individual layers along the *z*‐axis of the print.^[^ ^11^ ^]^ So far, none of the glass printing processes is capable of directly printing glass components with smooth surfaces below 10 nm. However, a great variety of novel applications like compact multilens objectives or free‐form coupling elements have emerged in the field of optics and photonics, sensing and analysis, which require high‐precision 3D structuring of optical materials.^[^ ^18^, ^19^, ^20^ ^]^

Two‐photon polymerization direct laser writing (DLW) has evolved as a 3D printing technology able to fabricate 3D microoptics with (sub‐)micrometer feature sizes and optically smooth surfaces.^[^ ^21^, ^22^ ^]^ Compared to SL printing, DLW uses two‐photon absorption, which locally confines the polymerizing voxel, allowing for higher resolutions and smoother surfaces. Although voxel speeds for DLW can be higher than for SL the small voxel size in combination with the serial nature of the process makes fabrication of higher volume components more time consuming compared to SL.^[^ ^23^ ^]^ While highest precision DLW printing is achieved with polymers, researchers have also explored routes to different material classes, such as ceramics or metals.^[^ ^24^, ^25^ ^]^ Nevertheless, glass is very often considered the desired material of choice for many applications, because of its superior material properties in terms of optical transparency and thermal, mechanical, and chemical resistance.

In this work, we demonstrate that fused silica glass can effectively be structured by DLW using a two‐photon curable silica nanocomposite resin achieving 3D resolution in the range of tens of micrometers and a surface roughness of 6 nm.

We used a liquid Glassomer silica nanocomposite material, consisting of amorphous silica nanoparticles with a mean diameter size of around 40 nm dispersed in a monomeric binder matrix. The basis of this nanocomposite was a material, which we previously described for stereolithography printing.^[^ ^11^ ^]^ However, these materials could not directly be used for high‐speed and high‐resolution DLW, because the curing speed was not sufficiently high and the transparency of the resins at the processing wavelength of 780 nm was only ≈76% which is too low for DLW. We therefore increased the chemical crosslink of the materials to allow for a sufficient curing speed and chemical stability. The crosslinker was chosen to match the refractive index of the binder matrix to the refractive index of fused silica glass resulting in a nanocomposite with the required high optical transparency of 91.6% at the processing wavelength of 780 nm (see **Figure** **1**b). To achieve the resin for two‐photon printing (R6‐3, see Figure 1c), the liquid nanocomposite is further equipped with an initiation system tailored to conduct efficient radical polymerization at the two‐photon printing conditions of the printer. Printing was performed using a Photonic Professional GT2 system (Nanoscribe GmbH), equipped with the 3D LF feature set, i.e., dip‐in configuration using a 10× NA 0.3 objective and a silicon substrate. The printing process results in a polymeric nanocomposite structure. Excess non‐polymerized material is removed in a development step by immersing the parts in methanol for 10 min to obtain the green part (see Figure 1d). The binder matrix is subsequently removed in a thermal debinding process at a maximum temperature of 600 °C resulting in a porous brown part. This brown part is sintered to a dense transparent fused silica glass employing a temperature profile with a maximum temperature of 1300 °C. The process from green part to fused silica glass is shown in Figure 1a. The heating protocol for thermal debinding and sintering is listed in Table S1 (Supporting Information). During the sintering process, the parts undergo a linear shrinkage of 26.7 ± 0.2%, as can be seen from the rod shown in Figure 3a which showed a shrinkage in height from 1.804 to 1.318 mm and in width from 660 to 485 µm.

**Figure 1:**
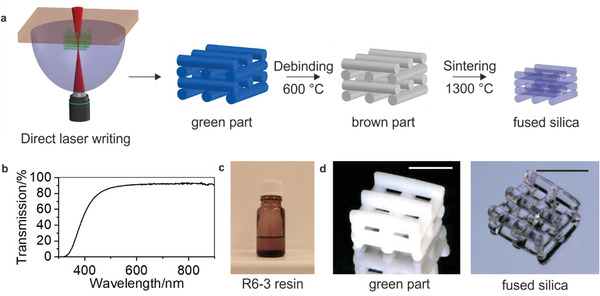
Direct laser writing of transparent fused silica glass. a) Schematic of the developed process for DLW of 3D fused silica microparts using silica nanocomposites. Glassomer nanocomposites consist of silica nanoparticles inside a photocurable binder matrix. The resin R6‐3 is polymerized and shaped using two‐photon DLW. The resulting polymerized nanocomposite is turned into transparent fused silica glass using thermal debinding and sintering. b) UV–vis spectrograph of silica nanocomposite without initiating system, showing a transmission of ≈91.7% at the illumination wavelength of 780 nm. c) Photograph of the R6‐3 nanocomposite resin. d) Logpile structure as green part and sintered fused silica glass part (scale bars: 500 µm).

We have fabricated a number of microstructures to demonstrate that DLW of silica nanocomposites allows shaping of fused silica glass structures with so far unseen precision, complexity, and low surface roughness. Figure 1d depicts an exemplary 3D logpile structure with a single rod width of 130 µm and a rod‐to‐rod distance of 145 µm (*xy*) and 75 µm (*z*). **Figure** **2**a,b shows an exemplary microrook with a height of ≈2 mm and pinnacles with a width of 200 µm. Figure 2c,d shows a sintered microfluidic filter element with a pore size of 55 µm. Pores with a minimum pore diameter of down to 14 µm could be successfully manufactured (Figure S2, Supporting Information). We further show that fused silica glass microoptical elements can be fabricated using this approach, demonstrated with the upright optical microlens in Figure 2e,f. To demonstrate the usage for the fabrication of optical components, we have printed three upright microlenses together and subsequently (after sintering on a silicon substrate), three polymeric Wigner–Seitz‐cell structures onto the same substrate (see Figure 2g). The front view of the lenses in Figure 2h shows the cell structures at their magnification, originating from the distance of the cell to the respective microlens. The focal length of such a lens was simulated to be about 790 µm for the configuration convex‐plano and 880 µm for the configuration plano‐convex. Simulation details are shown in Figure S4 (Supporting Information). The surface roughness of the sintered fused silica glass was characterized using a confocal microscope on an exemplary printed microlens array showing a low surface roughness of *R*_a_ ≈ 6.1 nm without the need of further postprocessing (see **Figure** **3**b). The shape accuracy (*S*_a_) for such a microlens printed on top of a substrate (Figure 3b) and the upright lens shown in Figure 2e were characterized using confocal microscopy to be 700 and 250 nm, respectively. The pseudo‐color images for *S*_a_ calculation are shown in Figure S3 (Supporting Information). Sintering of the microlenses on top of a non‐shrinking substrate results in an additional deviation because of an anisotropic shrinkage effect. The upright standing lens was printed on a pedestal, to account for the shrinking and allow for an isotropic shrinkage. The encountered deviation in this lens is characteristic for the here‐employed DLW printing parameters and is also found in high‐precision printing of polymers.^[^ ^26^ ^]^ Substantial reduction of such shape deviation typically requires dedicated printing protocols designed for the respective optical part. The refractive index of the sintered fused silica glass was measured to be *n*_D20_ = 1.4585 ± 0.0006, which is in good accordance with the refractive index of commercial fused silica glass with *n*_D20_ = 1.4585.^[^ ^27^ ^]^ We have further measured the optical transparency using UV–vis measurement of the sintered fused silica glass and compared it to commercial fused silica glass (see Figure S4, Supporting Information). As can be seen the sintered fused silica glass shows an equivalent high optical transparency.

**Figure 2:**
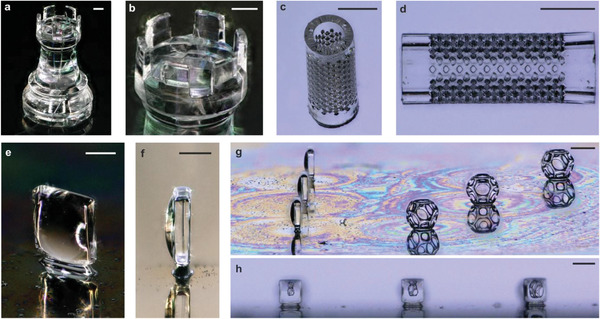
3D microstructuring of fused silica glass using DLW. a,b) Exemplary microrook with a height of ≈2 mm (scale bars: (b): 200 µm). c,d) Microfilter element with a pore size of 55 µm (scale bars: 500 µm). e) Fused silica glass upright lens (scale bar: 180 µm). f) Side view of the standing lens of (e) (scale bar: 190 µm). g) Three fused silica upright microlenses directly printed on a silicon substrate with three Wigner–Seitz cells printed in IP‐Q (scale bar: 400 µm). h) Front view through the three fused silica glass microlenses in (g) showing the cells at different distances (scale bar: 360 µm).

**Figure 3:**
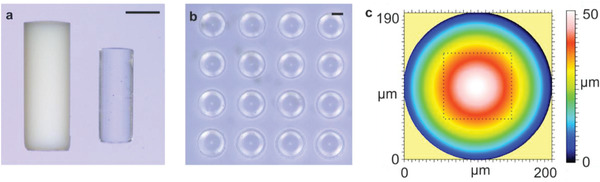
Characterization of sintered fused silica glass. a) Microscopy image of green part and sintered fused silica glass showing an isotropic shrinkage of 26.7% (scale bar: 485 µm). b) Microscopy image of a printed fused silica microlens array (scale bar: 100 µm). c) Confocal microscopy image of a single lens from the microlens array shown in (b). Roughness was measured within the highlighted area (dotted lines).

In summary, we developed a DLW process based on two‐photon polymerization for structuring transparent fused silica glass via a silica nanocomposite and subsequent thermal densification. Using this process, fused silica components with tens of micrometer resolution and low surface roughness were fabricated. The process allows the generation of optically smooth 3D fused silica glass components. This technique constitutes a major breakthrough in the fabrication of novel high‐resolution glass components pushing the boundaries of resolution, shape and design of freedom for many high‐performance applications including optics, photonics, functional and engineered surfaces as well as lab‐on‐a‐chip, life sciences, and biomedical engineering.

## Experimental Section

### Materials

The prototype resin R6‐3 (Nanoscribe GmbH, Germany) is a highly crosslinking photocurable silica Glassomer nanocomposite, with a solid loading of 32.5 vol% silica nanopowder in a photocurable binder matrix and an initiation system for optimum two‐photon printing performance. The binder matrix consisted of 40 vol% hydroxyethyl methacrylate (HEMA) and 60 vol% of the cross‐linker trimethylolpropane ethoxylate triacrylate. IP‐Q was obtained from Nanoscribe GmbH, Germany. Methanol was purchased from Sigma‐Aldrich, Germany. Isopropanol, used for cleaning of the objective and other printing equipment, was purchased from Sigma‐Aldrich, Germany. All substrates were obtained from Nanoscribe GmbH, Germany.

### Direct Laser Writing

3D objects were fabricated using the commercial lithography system Photonic Professional GT2 (Nanoscribe GmbH, Germany). Silicon substrates (3D LF DiLL, 25 × 25 × 0.725 mm, from Nanoscribe GmbH, Germany) were used. The substrates were activated by oxygen plasma to ensure efficient adhesion of the printed green part to the substrate during development. The resin R6‐3 was dropcast onto the activated substrate prior to printing. The objective 3D Large Feature DiLL 2PP 10×/0.3 (from Nanoscribe GmbH, Germany) was used for printing. Printing was performed using the following parameters: slicing 5 µm, hatching 1 µm, scan speed 100 mm s^−1^, and laser power 80%. For printing of the rook structure, splitting was necessary. Splitting values: mode: rectangular, block size *x*/*y*/*z*: 500/500/700 µm, shear: 15°, overlap *x*/*y*/*z*: 40/40/4 µm, block order: meander. After printing, excess resin was removed by immersing the prints in methanol for 10 min. The resulting green part was either removed from the substrate (logpile, rook, filter, rod) or left on the substrate (upright lens, microlens array) prior to heat treatment. Samples were removed from the substrate using a razorblade and plastic tweezers. Care must be taken during this step as green part structures are fragile and break easily. The Wigner–Seitz cells were printed using IP‐Q and the 3D SF solid recipe. The microlens array was printed on a fused silica substrate (3D SF DiLL, 25 × 25 × 0.7 mm, from Nanoscribe GmbH) which requires the interface to be found manually. Slicing of the microlens array structure was set to be 1 µm.

### Heat Treatment

Thermal debinding was done in an ashing furnace of type AAF (Carbolite/Gero, Germany). The debound parts were sintered in a high‐temperature tube furnace of type STF16/450 (Carbolite/Gero, Germany) at a temperature of 1300 °C for 2 h. The samples were sintered under vacuum at a pressure of 5 × 10^–2 ^mbar. The heating and cooling rate was 3 K min^–1^. The complete protocol for thermal debinding and sintering can be found in Table S1 (Supporting Information).

### Roughness/Shape Accuracy Measurement

Surface roughness was measured using a confocal microscope of type μsurf expert (purchased from Nanofocus, Germany). Surface roughness was further measured on a DLW‐printed fused silica glass sample using a white‐light interferometer of type NewView 9000 (purchased from Zygo, USA; see the Supporting Information).

### Optical Characterization

The optical transparency was measured using a UV–vis spectrometer of type Evolution 201 (purchased from Thermo Scientific, Germany). The refractive index was measured using a refractometer of type ATR‐L (purchased from Schmidt+Haensch, Germany) at a temperature of 25 °C at a wavelength of 589.3 nm. The focal length was simulated using a beam propagation software recently described.^[^ ^28^ ^]^ Input: a Gaussian beam with a mode field diameter of 150 µm, the measured shape of the lens (by confocal microscopy), and the refractive index of fused silica glass.

## Conflict of Interest

Glassomer and Nanoscribe will commerziallize the material developed and described in this paper.

## Supporting information

Supporting Information
