# Supplementary material for: Two‐Photon Polymerization of Nanocomposites for the Fabrication of Transparent Fused Silica Glass Microstructures
Source: Adv Mater. 2021 Jan 14;33(9):2006341. doi: 10.1002/adma.202006341 (PMC11469267; doi:10.1002/adma.202006341)
Supplement: Supplementary file 1 — Supporting Information [file ADMA-33-2006341-s001.pdf]

© 2021 Wiley-VCH GmbH

ADVANCED MATERIALS

Supporting Information

for *Adv. Mater.*, DOI: 10.1002/adma.202006341

Two-Photon Polymerization of Nanocomposites for the Fabrication of Transparent Fused Silica Glass Microstructures

Frederik Kotz, Alexander S. Quick, Patrick Risch, Tanja Martin, Tobias Hoose, Michael Thiel, Dorothea Helmer, and Bastian E. Rapp*

Supporting Information

Two-Photon Polymerization of Nanocomposites for the Fabrication of Transparent Fused Silica Glass Microstructures

Frederik Kotz, Alexander S. Quick, Patrick Risch, Tanja Martin, Tobias Hoose, Michael Thiel, Dorothea Helmer, Bastian E. Rapp*

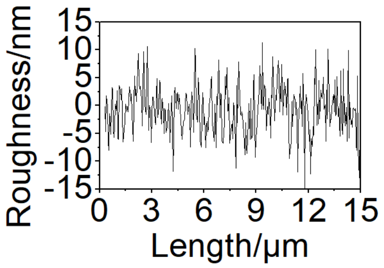

Figure S1: Roughness characterization of sintered Glassomer component in Figure S2 using white light interferometry showing a surface roughness of R_a ~ 1 nm.

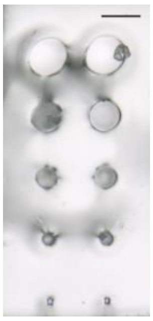

Figure S2: Minimum pore size in sintered Glassomer. The minimum achievable pore size was 14 µm (scale bar: 65 µm)

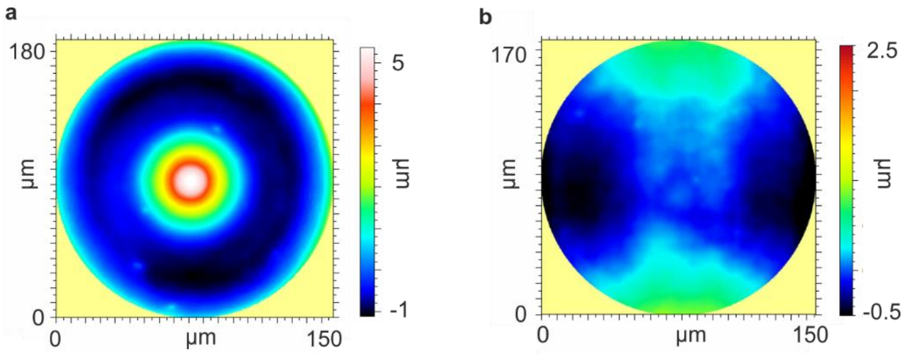

Figure S3: Pseudo-color image of a sintered microlenses. a) Sintering on top of a commercial fused silica glass slide results in a partially anisotropic shrinkage. The color mapping depicts the deviation of the produced shape from an ideal hemisphere. The S_a value was measured to be 700 nm. b) Sintering of the upright microlens on a compensationg pedestal from Fig. 2e results in an isotropic shrinkage. The S_a value was measured to be 250 nm. These deviations occurred already during the printing process.

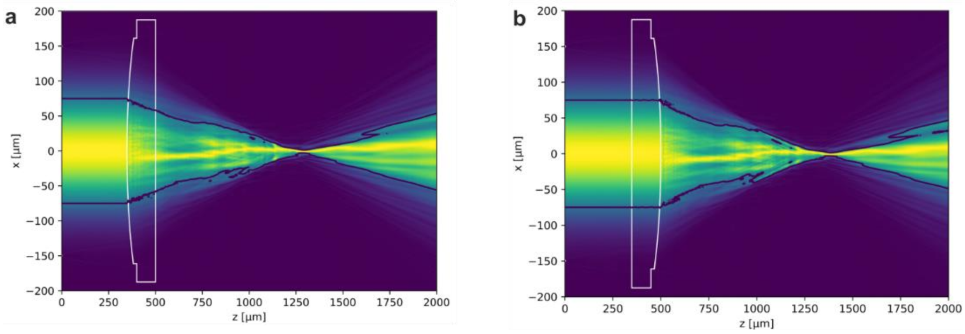

Figure S4: Simulation of focal length of the lens shown in Figure 2e based on beam propagation. Input: a Gaussian beam with a mode field diameter of 150 µm, a wavelength of 520 nm, and the refractive index of fused silica glass. a) For the configuration convex-plano the focal length is about 790 µm. b) For the configuration plano-convex the focal length is about 880 µm. The plotted color coding describes the normalized electrical field. The blue contour line shows the 1/e electrical field value of the gaussian beam. The lens is depicted by the white contour line.

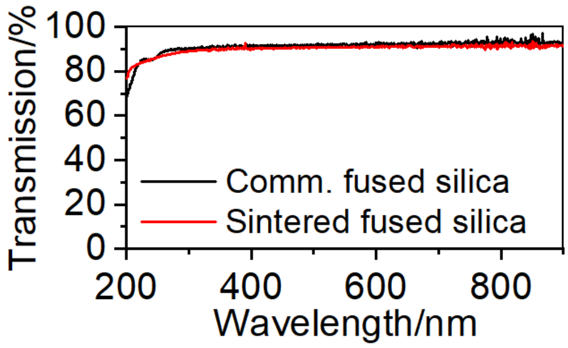

Figure S5: UV/Vis transmission spectra of sintered fused silica glass and a commercial fused silica substrate with a thickness of 1 mm.

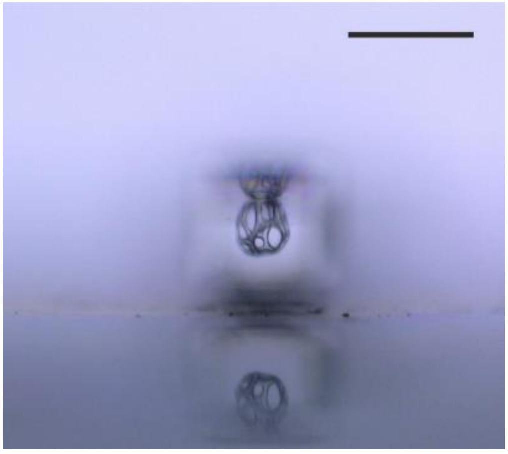

Figure S6: Magnification of an imaging demonstration for an upright lens shown in Figure 2h.

Thermal debinding and sintering of the Glassomer nanocomposites was done using the protocol shown in Table S1.

Table S1. Heat treatment for thermal debinding and sintering

Program	Temperature/°C	Heating rate/°C/min	Holding Phase/min
**Thermal Debinding**	150	0.5	120
	320	0.5	240
	600	0.5	120
	25	10	-
**Sintering**	1300	3	120
	25	3	-
